# The amniotic fluid proteome predicts imminent preterm delivery in asymptomatic women with a short cervix

**DOI:** 10.1038/s41598-022-15392-3

**Published:** 2022-07-11

**Authors:** Dereje W. Gudicha, Roberto Romero, Nardhy Gomez-Lopez, Jose Galaz, Gaurav Bhatti, Bogdan Done, Eunjung Jung, Dahiana M. Gallo, Mariachiara Bosco, Manaphat Suksai, Ramiro Diaz-Primera, Piya Chaemsaithong, Francesca Gotsch, Stanley M. Berry, Tinnakorn Chaiworapongsa, Adi L. Tarca

**Affiliations:** 1 Perinatology Research Branch, Division of Obstetrics and Maternal-Fetal Medicine, Division of Intramural Research, Eunice Kennedy Shriver National Institute of Child Health and Human Development, National Institutes of Health, U.S. Department of Health and Human Services Bethesda, MD, Detroit, MI USA; 2 Department of Obstetrics and Gynecology, Wayne State University School of Medicine, Detroit, MI USA; 3 Department of Obstetrics and Gynecology, University of Michigan, Ann Arbor, MI USA; 4 Department of Epidemiology and Biostatistics, Michigan State University, East Lansing, MI USA; 5 Center for Molecular Medicine and Genetics, Wayne State University, Detroit, MI USA; 6 Detroit Medical Center, Detroit, MI USA; 7 Department of Biochemistry, Microbiology and Immunology, Wayne State University School of Medicine, Detroit, MI USA; 8 Department of Computer Science, Wayne State University College of Engineering, Detroit, MI USA

**Keywords:** Computational biology and bioinformatics, Immunology, Systems biology, Biomarkers, Health care, Medical research, Molecular medicine, Risk factors

## Abstract

Preterm birth, the leading cause of perinatal morbidity and mortality, is associated with increased risk of short- and long-term adverse outcomes. For women identified as at risk for preterm birth attributable to a sonographic short cervix, the determination of imminent delivery is crucial for patient management. The current study aimed to identify amniotic fluid (AF) proteins that could predict imminent delivery in asymptomatic patients with a short cervix. This retrospective cohort study included women enrolled between May 2002 and September 2015 who were diagnosed with a sonographic short cervix (< 25 mm) at 16–32 weeks of gestation. Amniocenteses were performed to exclude intra-amniotic infection; none of the women included had clinical signs of infection or labor at the time of amniocentesis. An aptamer-based multiplex platform was used to profile 1310 AF proteins, and the differential protein abundance between women who delivered within two weeks from amniocentesis, and those who did not, was determined. The analysis included adjustment for quantitative cervical length and control of the false-positive rate at 10%. The area under the receiver operating characteristic curve was calculated to determine whether protein abundance in combination with cervical length improved the prediction of imminent preterm delivery as compared to cervical length alone. Of the 1,310 proteins profiled in AF, 17 were differentially abundant in women destined to deliver within two weeks of amniocentesis independently of the cervical length (adjusted p-value < 0.10). The decreased abundance of SNAP25 and the increased abundance of GPI, PTPN11, OLR1, ENO1, GAPDH, CHI3L1, RETN, CSF3, LCN2, CXCL1, CXCL8, PGLYRP1, LDHB, IL6, MMP8, and PRTN3 were associated with an increased risk of imminent delivery (odds ratio > 1.5 for each). The sensitivity at a 10% false-positive rate for the prediction of imminent delivery by a quantitative cervical length alone was 38%, yet it increased to 79% when combined with the abundance of four AF proteins (CXCL8, SNAP25, PTPN11, and MMP8). Neutrophil-mediated immunity, neutrophil activation, granulocyte activation, myeloid leukocyte activation, and myeloid leukocyte-mediated immunity were biological processes impacted by protein dysregulation in women destined to deliver within two weeks of diagnosis. The combination of AF protein abundance and quantitative cervical length improves prediction of the timing of delivery compared to cervical length alone, among women with a sonographic short cervix.

## Introduction

Preterm birth, the leading cause of perinatal morbidity and mortality^1–7^, is associated with an increased risk of short- and long-term health outcomes for neonates who survive^8–13^. Worldwide, preterm birth affects about 15 million babies annually, which accounts for the 11% global preterm birth rate^3, 14, 15^. In the United States, the rate of preterm birth has been approximately 10% since 2018, and this number remains high as compared to the rate observed in other developed countries^3, 16, 17^. The rate of preterm birth is even higher in several developing countries^3, 4, 18^ and contributes to substantial costs related to healthcare services^19–22^.

The identification of women at risk of preterm birth is central to the development of effective, preventive interventions aimed to reduce the potential negative effects at birth or later in life^7, 23–26^. Although various strategies to screen women at risk of preterm birth have been proposed by investigators, more accurate methods are yet to be developed, given the multifactorial causes leading to this syndrome^7, 27, 28^. Risk factors for preterm birth include advanced maternal age^29^, greater maternal body mass index^30, 31^, substance use during pregnancy (tobacco or alcohol use)^32, 33^, exposure to violence (physical or emotional)^34–36^, and psychosocial stress^37, 38^. In addition, clinical and obstetrical characteristics, such as a history of previous preterm birth^39–41^, gestational diabetes and chronic hypertension^42^, short inter-pregnancy interval^43, 44^, infection and inflammation^45–50^, genetic factors^51–54^, and environmental pollutants^55–59^, have also been linked to an increased risk of preterm birth. Race/ethnicity-related disparities in preterm birth rates were also reported^60–62^.

The traditional approach in the screening of imminent preterm birth involves a combination of maternal and obstetrical characteristics^63–65^. However, the detection rate of this approach is low (sensitivity ~ 20% and positive predictive value ~ 30%)^63^. Molecular biomarkers, such as fetal fibronectin in cervico-vaginal secretions^66–69^ and increased concentrations of interleukin (IL)-6 in amniotic fluid (AF)^70–73^ have also been associated with a higher risk of preterm birth. Novel proteomics platforms and bioinformatics algorithms have enabled a refined characterization of the AF proteome^27, 74–87^ for the prediction of several pathological conditions in pregnancy^88–90^, including preterm birth^23, 91–94^. For example, Lee et al.^95^ showed that AF cytokines and matrix metalloproteinases in combination with clinical risk factors improve the prediction of early preterm birth compared to a single protein or each clinical factor alone. Other investigators suggested a combination of multiple markers that include those profiled in AF and cervical fluid^96–98^.

In addition to biochemical markers, a powerful predictor of preterm birth is a transvaginal sonographic short cervix^99–106^, and women at risk can benefit from vaginal progesterone treatment^107–111^. We have proposed that the sensitivity of cervical length screening can be further improved by using a customized approach that accounts for maternal characteristics (weight, height, and parity) and exact gestational age at screening^112^. For women identified as at risk attributable to a short cervix at any time during the second or third trimester, it would be important, to know whether delivery is imminent. Therefore, in this study, we sought to identify AF proteins that can predict imminent preterm delivery in women with a sonographic short cervix.

## Materials and methods

### Study population and design

This was a retrospective analysis of data collected from pregnant women who were enrolled in a longitudinal biomarker study involving universal cervical length measurement at the Center for Advanced Obstetrical Care and Research of the Perinatology Research Branch of the *Eunice Kennedy Shriver* National Institute of Child Health and Human Development (NICHD), the Detroit Medical Center, and Wayne State University (Detroit, MI, USA). Briefly, pregnant women were enrolled between 6 and 22 weeks of gestation and followed until delivery. Exclusion criteria included women who had the following diagnosis at the time of recruitment: preterm labor, preterm premature rupture of the membranes, preeclampsia, fetal growth restriction, active vaginal bleeding, multifetal gestation, and serious medical illness such as renal insufficiency, congestive heart disease, chronic respiratory insufficiency, etc. The protocol called for sonographic cervical length in the midtrimester followed by measurements every four weeks until 24 weeks of gestation, then every two weeks until delivery. When the cervical length measured was 25 mm or less, patients were sent to the obstetrical triage area for evaluation and counseling regarding risks of intra-amniotic infection/inflammation and preterm birth. Treatment with antibiotics were shown successful in a subset of patients with cervical insufficiency and intra-amniotic inflammation^113, 114^. The decision to offer amniocentesis was at the discretion of treating physicians.

The techniques for sonographic assessments of the short cervix and amniotic fluid sample collection were described in previous reports^112, 115, 116^. We retrospectively selected women with a singleton pregnancy and a sonographic short cervix (≤ 25 mm) between 16 and 32 weeks of gestation who had a transabdominal amniocentesis performed within two days of the cervical length measurement. Only cases without clinical signs of infection or labor at the time of amniocentesis were included. The primary indication for amniocentesis in this group of asymptomatic patients was to rule out intra-amniotic infection/inflammation due to a short cervix. For a subset of these patients, fetal karyotype and fetal lung maturity testing were also performed. Additional exclusion criteria for this study were labor induction for any reasons within two weeks of the amniocentesis, a positive AF culture for micro-organisms, abnormal fetal karyotypes or chromosomal microarray, and structural fetal anomalies. Participants in the study were recruited between May 2002 and September 2015, and all provided informed written consent prior to the collection of samples and images. The use of the data collected (demographic or clinical information, images, and samples) for research purposes was approved by the Human Investigation Committee of Wayne State University and the Institutional Review Board of NICHD. All methods were performed in accordance with relevant guidelines and regulations.

### Proteomics profiling

The concentration of 1,310 proteins in AF samples was quantified by using the SOMAmer (Slow Off-rate Modified Aptamers) platform and its reagents, and proteomics profiling was performed by Somalogic, Inc. (Boulder, CO, USA), as described in previous publications^117–119^. Briefly, AF samples were diluted and then incubated with a mixture of SOMAmers on streptavidin-coated beads. Next, the beads were washed to remove all unbounded proteins and other matrix constituents, and proteins that remained bound to their cognate SOMAmer reagents were tagged with an NHS-biotin reagent. Pure cognate-SOMAmer complexes and unbound SOMAmer reagents were released from streptavidin beads by ultraviolet light that cleaved the photo-cleavable linker used to quantitate protein. The photo-cleavage eluate was separated from the beads and then incubated with a second streptavidin-coated bead. The free SOMAmer reagents were then removed during subsequent washing steps. In the final elution step, protein-bound SOMAmer reagents were released from their cognate proteins by using denaturing conditions. SOMAmer reagents were then quantified by hybridization to custom DNA microarrays. The cyanine-3 signal from the SOMAmer reagent was detected on the microarrays.

## Statistical analyses

### Demographic data analysis

The demographic and clinical characteristics were compared by using Wilcoxon-signed rank tests for continuous variables and Fisher’s exact tests for categorical variables. A p-value < 0.05 for the differences between the groups was considered statistically significant.

### Proteomic data analysis

To identify AF protein dysregulation that can be informative about the timing of delivery, we fit linear models on log_2_-transformed relative fluorescence unit (RFU) values, using an explanatory variable for the delivery group: within two weeks (imminent delivery) vs. greater than two weeks until delivery. To account for the residual information that quantitative cervical length measurement may provide, we included cervical length as a covariate in the linear models. The significance of the group differences was assessed via moderated t-tests. An advantage of the moderate t-test, as contrasted with the standard t-test, is that it borrows information across the different proteins to derive more robust estimates of protein data variance^120^, and it has also been shown to improve the selection of predictors for omics data-based multi-variate predictive models^121–123^. Protein p-values were adjusted for multiple testing, and the false-positive discovery rate was controlled at the 10% level (q-value < 0.1). The linear models were fit by using the *limma* package in R/Bioconductor^124^.

Logistic regression models were also implemented to determine the odds of imminent delivery associated with a two-fold change in protein abundance, while adjusting for cervical length. The area under the receiver operating characteristic curve (AUC) was calculated to determine whether protein data improves the prediction of imminent delivery as compared to cervical length alone. Combinations of up to four proteins were also evaluated by using multivariate logistic regression and the AUC was determined. In addition, Kaplan–Meier survival curves based on the interval from amniocentesis to delivery were compared between patients with a risk score above and those with a risk score below a cut-off value corresponding to a 10% false-positive rate.

To identify biological processes overrepresented in the list of proteins associated with imminent delivery, we performed a Gene Ontology (GO) enrichment analysis with the *clusterProfiler package*^125^ in R/Bioconductor*.* The enrichment analyses also involved control for the false-positive discovery rate at 10% level. Visualization of the abundance of significant protein profiles was performed by using the *heatmap* function in the *ComplexHeatmap* package^126^.

## Results

### Demographic and clinical characteristics

The study included 90 women diagnosed with a sonographic short cervix (< 25 mm) during the second or third trimester. Of this group, 24 women delivered within two weeks from amniocentesis (n = 24) and the remaining 66 women delivered after two weeks from amniocentesis (n = 66). The characteristics of the study population are shown in Table 1, and the gestational ages at amniocentesis in both groups are depicted in Figure S1. The two groups were similar with respect to gestational age at amniocentesis, maternal age, weight, body mass index, race, parity, and history of preterm birth (p > 0.05). However, cervical length (median 5 vs. 15 mm, p < 0.001), gestational age at delivery (median 24.2 vs. 38.7 weeks, p < 0.001), and birthweight (median 651 vs. 2985 g, p < 0.001) were lower in women who delivered within two weeks compared to those who did not. In addition, neonates delivered within two weeks from amniocentesis had a significantly higher frequency of an Apgar score < 7 at 5 min [56.57% (13/23) vs. 7.7% (5/65), p < 0.001] and of admission to a neonatal intensive care unit [62.5% (15/24) vs. 13.6% (9/66), p < 0.001], compared to neonates whose delivery occurred after two weeks from amniocentesis. The presence of severe acute histologic chorioamnionitis was also more frequent among the women who delivered within two weeks of amniocentesis [81.2% (13/16) vs. 8.7% (4/46), p < 0.001]. Among the women who delivered within two weeks of amniocentesis, 50% (12/24) had intra-amniotic inflammation, indicated by an elevated AF concentration of IL-6 (IL-6 ≥ 2.6 ng/mL) compared to 7.6% (5/66) of those who delivered more than two weeks after an amniocentesis. Of note, the concentrations of IL-6 measured by ELISA were highly correlated to the relative fluorescence measures derived by the aptamer platform (Spearman’s correlation 0.89, p < 0.01).

**Table 1:** Demographic and clinical characteristics of the study population.

Characteristics	Delivery at ≤ 2 weeks (n = 24)	Delivery at > 2 weeks (n = 66)	P value
Age	22.5 (19–32.2)	24 (21–26)	0.993
Height (cm)	160 (154.3–163.2)	162.6 (157.5–167.6)	0.047
Weight (kg)	69.2 (61–78.9)	77.1 (57.9–89.8)	0.404
Body mass index	28.7 (23.7–30.6)	28.2 (21.6–34.2)	0.898
Race (African–American)	87.5 (21/24)	80.3 (53/66)	0.544
Tobacco use	12.5 (3/24)	22.7 (15/66)	0.379
Alcohol use	0 (0/24)	3.1 (2/64)	
Nulliparous	54.2 (13/24)	42.4 (28/66)	0.348
History of preterm birth	29.2 (7/24)	24.2 (16/66)	0.785
Cervical length (mm)	5.5 (0–10.8)	15 (10–19.8)	0.001
Cervical dilation (cm)	1.00 (0.00–1.75)^a^	0.5 (0.00–1.00)^b^	0.07
Progesterone treatment	4.2 (1/24)	16.7 (11/66)	0.17
**Indications for amniocentesis**			
Detection of intra-amniotic infection/inflammation	95.8 (23/24)	98.5 (65/66)	0.464
Karyotyping	25 (6/24)	6.1 (4/66)	0.02
Polyhydroamnios	0 (0/24)	1.5 (1/66)	1
Fetal lung maturity	4.2 (1/24)	0 (0/66)	0.267
Gestational age at amniocentesis (weeks)	23 (20.4–25.5)	24.5 (22.2–27.6)	0.081
Gestational age at cervical length measurement (weeks)	23 (20.4–25.5)	24.5 (22.1–27.6)	0.082
Gestational age at delivery (weeks)	24.2 (21.5–27)	38.7 (36.2–39.4)	< 0.001
Preterm delivery	100 (24/24)	27.3 (18/66)	< 0.001
Baby weight (g)	650.5 (428.2–945)	2985 (2387–3364)	< 0.001
Cesarean delivery	12.5 (3/24)	24.2 (16/66)	0.381
Fetal sex (male)	43.5 (10/23)	60 (39/65)	0.223
Apgar score < 7 at 5 min after delivery	56.5 (13/23)	7.7 (5/65)	< 0.001
NICU Admission	62.5 (15/24)	13.6 (9/66)	< 0.001
Severe histologic chorioamnionitis	81.2 (13/16)	8.7 (4/46)	< 0.001
Severe funisitis	15.4 (2/13)	2.2 (1/45)	0.123
IL-6 ≥ 2.6 ng/mL	50 (12/24)	7.6 (5/66)	< 0.001

Data are presented as median (IQR) for continuous and % (n/N) for categorical variables. P-values calculated by Wilcoxon-signed rank tests and Fisher’s exact tests. Note, data were missing for select variables presented in the Table. NICU: neonatal intensive care unit.

^a^Missing one datum.

^b^Missing 7 data points.

### Differential protein abundance predictive of preterm delivery within two weeks from amniocentesis

**Figure 1:**
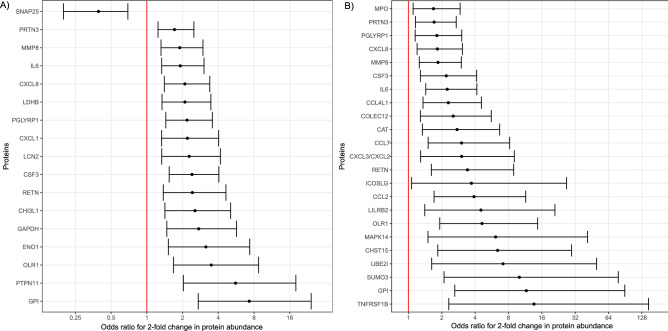
Odds ratio (and 95% confidence intervals) for the association between amniotic fluid proteins and imminent delivery. (**A**) Odds ratios for delivery within two weeks and (**B**) within one week. Odds ratios are adjusted for quantitative cervical length. The odd-ratios are calculated for a two-fold change in protein abundance. Alpha-enolase (ENO1), C–C motif chemokine 2 (CCL2), C–C motif chemokine 4-like (CCL4L1), C–C motif chemokine 7 (CCL7), C-X-C motif ligand 8 (CXCL8), Carbohydrate sulfotransferase 15 (CHST15), Catalase (CAT), Chitinase-3-like protein 1 (CHI3L1), Collectin-12 (COLEC12), Glucose-6-phosphate isomerase (GPI), Glyceraldehyde-3-phosphate dehydrogenase (GAPDH), Granulocyte colony-stimulating factor (CSF3), Gro-beta/gamma (CXCL3/CXCL2), Growth-regulated alpha protein (CXCL1), ICOS ligand (ICOSLG), Interleukin-6 (IL6), L-lactate dehydrogenase B chain (LDHB), Leukocyte immunoglobulin-like receptor subfamily B member 2 (LILRB2), Matrix Metalloproteinase-8 (MMP8), Mitogen-activated protein kinase 14 (MAPK14), Myeloperoxidase (MPO), Neutrophil gelatinase-associated lipocalin (LCN2), Oxidized low-density lipoprotein receptor 1 (OLR1), Proteinase 3 (PRTN3), Peptidoglycan recognition protein 1 (PGLYRP1), Resistin (RETN), Small ubiquitin-related modifier 3 (SUMO3), SUMO-conjugating enzyme UBC9 (UBE2I), Synaptosomal-associated protein 25 (SNAP25), Tumor necrosis factor receptor superfamily member 1B (TNFRSF1B), Tyrosine-protein phosphatase non-receptor type 11 (PTPN11).

Of the 1,310 proteins profiled in AF, 17 were differentially abundant in women destined to deliver within two weeks of amniocentesis, independently of the cervical length (adjusted p-value < 0.10). A higher abundance of Synaptosome Associated Protein 25 (SNAP25) in AF was associated with lower odds of an earlier delivery (adjusted odds ratio [OR] = 0.39) (Fig. 1A). By contrast, the risk of preterm birth within two weeks of amniocentesis increased with the higher abundance of the following proteins: Glucose-6-Phosphate Isomerase (GPI), Protein Tyrosine Phosphatase Non-receptor type 11 (PTPN11), Oxidized Low-density Lipoprotein Receptor 1 (OLR1), Enolase 1 (ENO1), Glyceraldehyde 3-Phosphate Dehydrogenase (GAPDH), Chitinase-3-like protein 1 (CHI3L1), Resistin (RETN), Colony-Stimulating Factor 3 (CSF3), Neutrophil gelatinase-associated lipocalin-2 (LCN2), C-X-C motif ligand 1 (CXCL1) and C-X-C motif ligand 8 (CXCL8), Peptidoglycan Recognition Protein 1 (PGLYRP1), Lactate Dehydrogenase B (LDHB), IL6, Matrix Metalloproteinase-8 (MMP8), and Proteinase 3 (PRTN3) (adjusted OR > 1.5). Of note, for all 17 proteins the significance p-value would be < 0.05 after adjusting for the secondary indication of amniocentesis, i.e. karyotype testing, which was slightly more frequent in women who delivered with two weeks (Table 1). This suggests that this confounding covariate was not a driver of the differential protein abundance observed herein. Therefore, we have attributed the proteomic differences observed to the pathophysiology leading to delivery within two weeks from amniocentesis.

### Differential protein abundance predictive of delivery within one week from amniocentesis

When comparing the protein abundance between women who delivered within one week from the amniocentesis (n = 9) to the group of women who delivered after one week (n = 81), we have identified 23 proteins with significant differential abundance after controlling the false discovery rate at the 10% level (q < 0.1). The cervical length adjusted ORs for the association of between a two-fold change in protein abundance and delivery within one week from amniocentesis are presented in Fig. 1B. Of note, among the 23 proteins that had higher abundance in the group of women who delivered within one week, nine were also identified as increased in women who delivered within two weeks from amniocentesis. Moreover, the point estimates of odds ratios for delivery with One week were larger than those for delivery within two weeks, suggesting a dose response relation between the timing of delivery and protein abundance changes.

### Prediction of delivery within two weeks from amniocentesis by cervical length and amniotic fluid proteins

**Figure 2:**
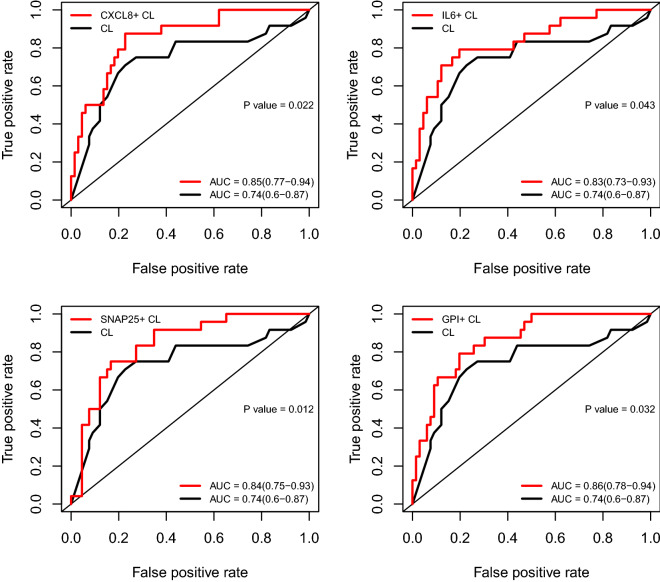
ROC curves for predicting imminent delivery. Each panel compares prediction by cervical length alone and models that combine cervical length with protein abundance. AUC: area under the ROC curve and 95% confidence interval. CL: cervical length.

Although all women had an amniocentesis after diagnosis with a short cervix, the exact cervical length (quantitative assessment) was still predictive of delivery within two weeks from amniocentesis, and shorter cervical lengths were associated with increased risk (AUC = 0.74) (Fig. 2). The addition of data from one protein at a time led to improvements in the AUC that ranged from 4% to 12% depending on the specific protein (Fig. 2). The greatest improvement in the AUC statistic, compared to cervical length alone, was noted for GPI (AUC 0.86 vs 0.74), followed by CSF3, CXCL8, SNAP25, GAPDH, PGLYRP1, IL6, OLR1, and LDHB (p < 0.05 for all). Of note, the predictive value of the combination of cervical length and ELISA-based IL-6 (AUC = 0.8) was similar to that of cervical length and aptamers-based multiplex IL-6 (AUC = 0.83) (Figure S2).

**Figure 3:**
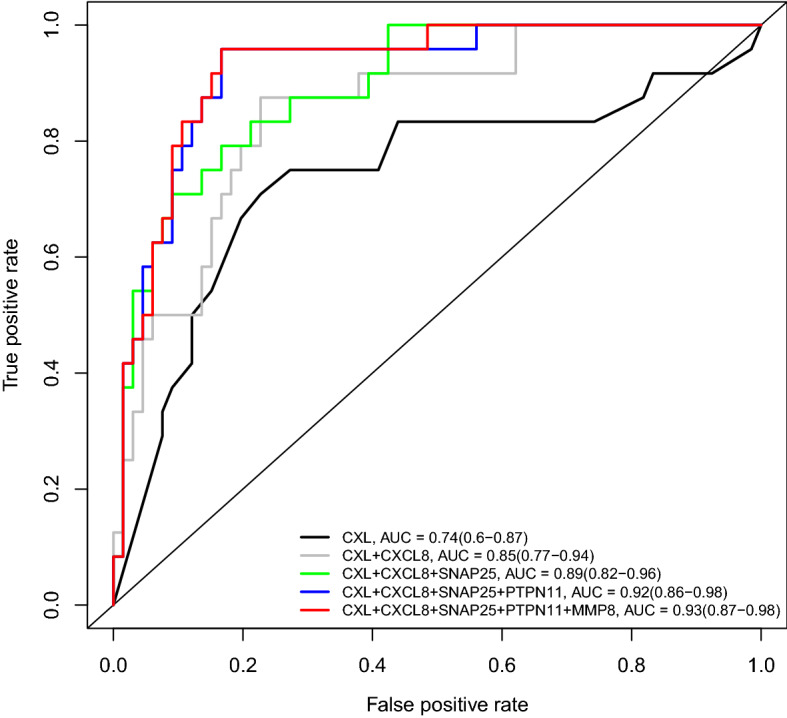
Combinations of multiple proteins for improving prediction of imminent delivery. AUC: area under the ROC curve and 95% confidence interval. CL: cervical length.

**Figure 4:**
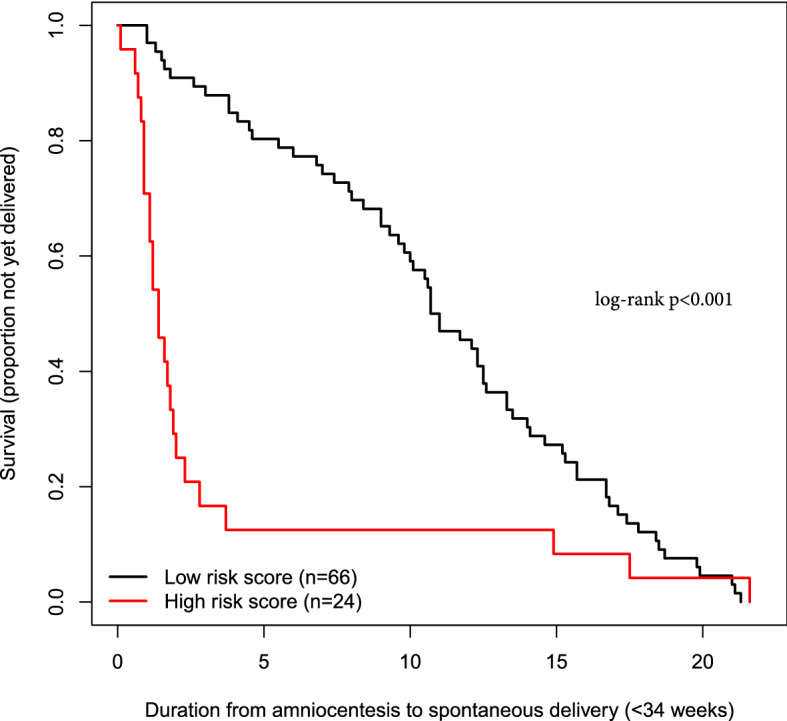
Kaplan–Meier survival curves for patients with a risk score above and those with a risk score below the cut-off value corresponding to a 10% false positive rate. The risk scores are determined by quantitative cervical length and four proteins (CXCL8, SNAP25, PTPN11, MMP8).

Combinations of the quantitative cervical length with up to four proteins further increased performance, reaching an AUC = 0.93 for the combination of CXCL8, MMP8, SNAP25, and PTPN11 (Fig. 3). The sensitivity, at a 10% false-positive rate, for the prediction of imminent delivery by the quantitative cervical length alone was 38%, yet it increased to 79% in combination with these four proteins. The Kaplan–Meier survival curves comparing duration to delivery between patients with a risk score above and those with a risk score below the risk cut-off value corresponding to a 10% false-positive rate are shown in Fig. 4. Patients with a risk score above the 10% false-positive rate cut-off value had a significantly shorter time to delivery compared to those with a risk score below the cut-off value [median: 1.4 (1.1–2.0) vs. 10.9 (9.8–13.3) weeks; log-rank p < 0.001].

**Figure 5:**
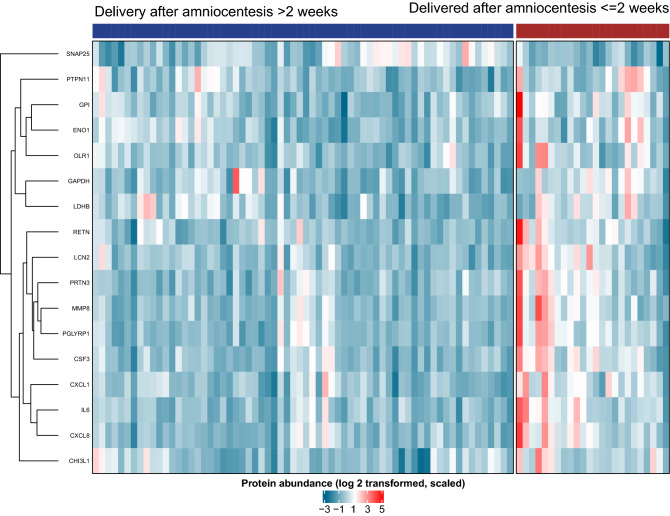
Clustered heatmap of protein data. The 17 proteins that were dysregulated in women destined for imminent delivery independent of cervical length were clustered using hierarchical clustering with correlation distance. Higher abundance of amniotic fluid protein levels is represented by a darker red color.

Given the similar predictive value among several proteins, we applied cluster analysis and identified two main sets of proteins with higher abundance in pregnant women destined to deliver within two weeks of amniocentesis (Fig. 5). Cluster #1 was dominated by pro-inflammatory cytokines, including some previously associated with a high risk of preterm birth: MMP8, PGYRP1, PRTN3, CSF3, LCN2, RETN, IL6, CXCL8, CXCL1, and CHI3L1. Member proteins of cluster #2 were ENO1, GPI, OLR1, PTPN11, LDHB, and GAPDH. One protein, SNAP25, formed a cluster by itself, as it was negatively correlated with the risk of imminent delivery.

### Gene ontology biological processes associated with imminent delivery

**Figure 6:**
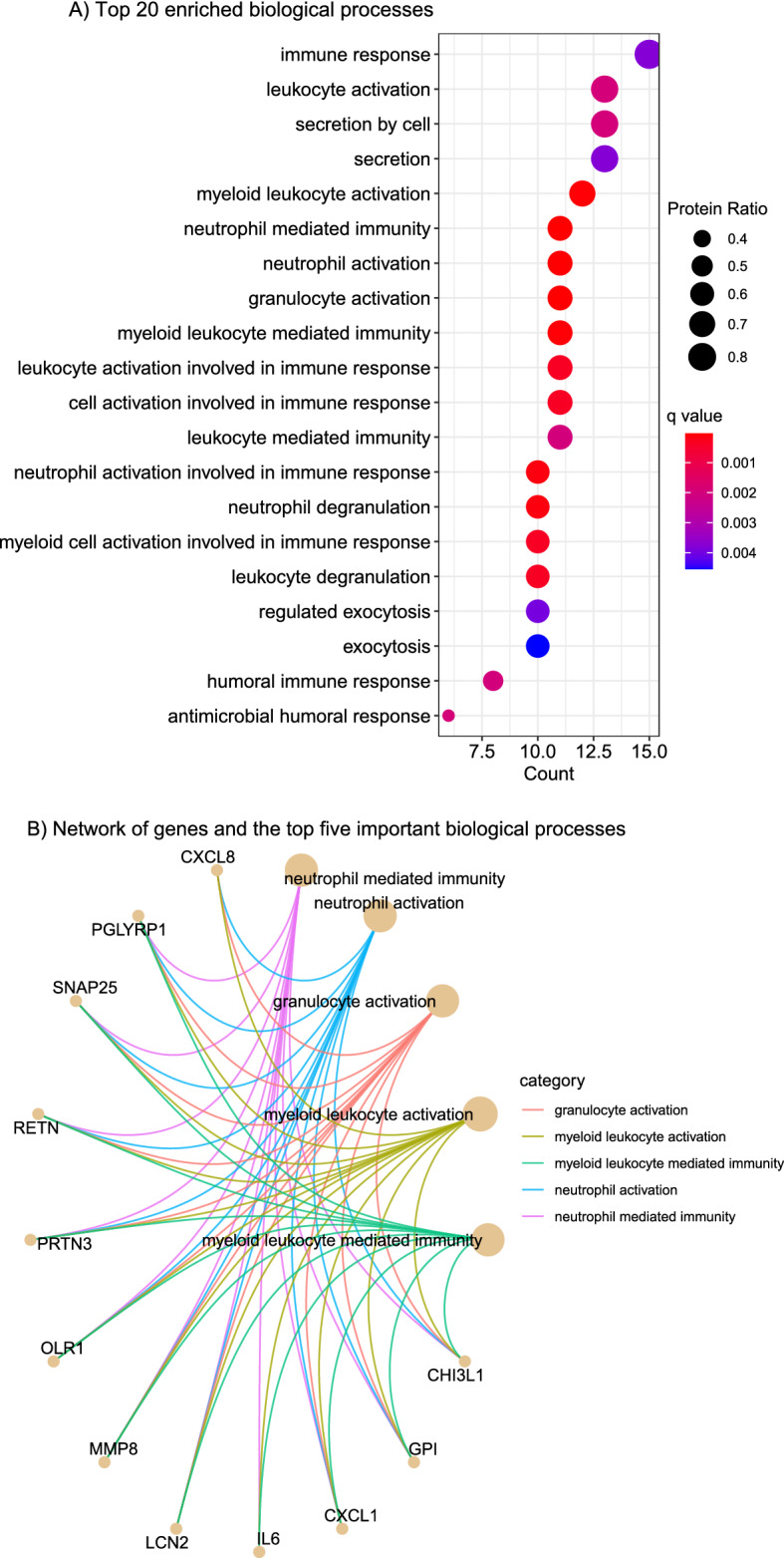
Gene ontology enrichment analysis of amniotic fluid proteins dysregulated with imminent delivery. Top 20 enriched biological processes (**A**) and a network representation of proteins involved in the top five enriched biological processes (**B**). Protein Ratio represents the number of proteins significantly dysregulated per the total number of proteins in a particular biological process. Darker red/blue indicate high/low enrichment, respectively. The size of the dots corresponds to the protein ratio represented by each biological process.

Enrichment analysis identified 23 biological processes associated with earlier preterm delivery after amniocentesis (q < 0.05, Table S1), which included neutrophil-mediated immunity, neutrophil activation, granulocyte activation, myeloid leukocyte activation, and myeloid leukocyte-mediated immunity (Fig. 6A,B).

## Discussion

### Principal findings of the study

(1) The AF proteins predicted imminent preterm delivery beyond what was previously possible when using only quantitative cervical length in women with a short cervix, a group already considered at risk for preterm birth (AUC = 0.93 for a combination of four proteins vs AUC = 0.74 for quantitative cervical length alone); (2) the sensitivity at a fixed false-positive rate of 10% for prediction of delivery within two weeks by a short cervix alone was 38%, yet it increased to 79% in combination with up to four AF proteins; and (3) neutrophil-mediated immunity, neutrophil activation, granulocyte activation, myeloid leukocyte activation, and myeloid leukocyte-mediated immunity were among the top biological processes associated with differentially abundant proteins in women who delivered within two weeks from an amniocentesis with a diagnosis of a short cervix.

### Our findings in the context of what is already known

Herein, we have assessed the value of AF proteins for the prediction of imminent delivery in pregnant women with a sonographic short cervix. Typically defined as cervical length < 25 mm, a short cervix was shown to be associated with a higher risk of preterm birth than those with a long cervix at any time during preterm gestation. For the 8–31 weeks interval, the 25 mm cut-off value was more extreme (lower) than the 10th percentile among asymptomatic women with term delivery in this population^112^. Studies from our group have shown that the rate of intra-amniotic infection and inflammation are substantial among women with a short cervix^115, 127, 128^. Women with a short cervix are already at risk for preterm birth; hence, it is important for patient management to distinguish those destined for imminent delivery. For example, women at risk of delivery within one week from amniocentesis may benefit from the administration of antenatal steroids to improve fetal lung maturity. A unique feature of this study, which confers predictive value, is that the data had been collected prior to any eventual symptoms of preterm labor.

Previous studies have reported that an elevated concentration of IL-6 in the maternal circulation increases the risk for preterm birth^94, 98, 129–131^. Intra-amniotic inflammation, defined as IL-6 ≥ 2.6 ng/mL^71^, is a known risk factor for preterm labor and delivery^70, 72, 91, 116, 132–136^. Increased concentration of IL-6 in cervico-vaginal fluid has also been implicated in women who delivered preterm^137, 138^. Similarly, a sonographic short cervix is also a risk factor for preterm delivery^116, 139^. However, few studies have assessed the value of combining AF IL-6 concentrations with cervical length measurement for the prediction of spontaneous preterm birth^97^. In the current study, we demonstrated that the AF IL-6 concentration adds predictive value to the quantitative cervical length for the prediction of imminent preterm birth in asymptomatic pregnant women with a sonographic short cervix. The same finding holds for MMP8, which is in agreement with previous studies that have linked MMP8 to intra-amniotic infection/inflammation^91, 135, 140–144^, a causal pathway leading to preterm birth^145–156^.

Among the family of C-X-C motif chemokines, we observed a significant increase of CXCL1 and CXCL8 levels in patients destined to deliver within two weeks of amniocentesis. The most predictive of these proteins, CXCL8, is also known as IL-8^157, 158^. We have shown that an abundance of CXCL8 in AF combined with quantitative cervical length improves the prediction of preterm birth as compared to cervical length alone (AUC = 0.85 vs. 0.74, p = 0.022). Several prior studies have related an increased abundance of CXCL8 to an activation of the innate immune system in response to microbial infection/inflammation^48, 50, 159–161^, while others have argued that such elevation of CXCL8 is physiological as well, resulting from molecular changes in preparation for labor^162–165^. Of note, increased CXCL8 was also reported in cervico-vaginal fluid of women with preterm delivery^166^.

The use of multiple biomarkers seems imperative in the overall goal to improve the prediction of preterm birth, given the heterogeneity of these conditions and the multiple causal pathways^95–98^. In line with these studies, we combined quantitative cervical length with multiple AF proteins (CXCL8, MMP8, PTPN11, and SNAP25) and found a significant improvement in the AUC (AUC = 0.93 for the combined markers vs. AUC = 0.74 for cervical length alone, p = 0.006).

### A possible role for neutrophil-mediated immunity in the intra-amniotic inflammatory response observed in pregnant women diagnosed with a short cervix

Neutrophils represent a primary cellular component of innate immunity that protects against microorganisms invading the amniotic cavity through an array of host defense mechanisms^167^, which may include phagocytosis^168^, the release of antimicrobial products and cytokines^75, 169–178^, and the formation of neutrophil extracellular traps^178–180^. Yet, neutrophils also form a physiological component of the AF cellular repertoire throughout pregnancy^181^; therefore, they are present in women diagnosed with a short cervix^182^. Herein, we found that biological processes, such as neutrophil-mediated immunity, neutrophil activation, granulocyte activation, myeloid leukocyte activation, and myeloid leukocyte-mediated immunity, were impacted by protein differential abundance in women who delivered within two weeks of the diagnosis of a short cervix. This finding suggests that AF neutrophils may undergo enhanced activation in women with a short cervix destined to deliver earlier preterm, either as a mechanism in response to bacterial products or “danger signal” in cases of sterile intra-amniotic inflammation^115^. However, further investigation is required to elucidate the participation of AF neutrophils in the inflammatory processes leading to earlier preterm delivery in women with a short cervix.

### Strength and limitations

This is the first study providing a comprehensive evaluation of AF proteins for the prediction of imminent delivery among asymptomatic pregnant women diagnosed with a sonographic short cervix. Some of the AF proteins we identified in the present study (GPI, PTRN11, OLR1, ENO1, GAPDH, CHI3L1, CSF3, LCN2, PGLYRP1, LDHB, PRTN3, and SNAP25) have not been widely explored in previous studies; therefore, they could provide additional insight into the discovery of biomarkers for further understanding of the pathophysiologic pathways leading to preterm birth^23^. Furthermore, the results of this study contribute to the growing interest in the use of multiple markers to predict preterm birth^183^. Our study demonstrates that when an asymptomatic patient presents with a sonographic short cervix between 16 and 32 weeks of gestation, specific AF proteins provide additional predictive power for identifying women at risk of imminent delivery (e.g. within 1 or 2 weeks), relative to cervical length alone. Limitations of this study are attributable to the timing of cervical length assessment and amniocentesis being within two days of each other, the limited power for assessing multi-variate prediction of delivery within one week of amniocentesis, and missing detailed obstetrical history such as type of prior preterm term birth and cervical surgery.

## Conclusions

Amniotic fluid protein abundance is predictive of imminent delivery among asymptomatic women with a sonographic short cervix. The combination of AF proteins and quantitative cervical length measurement provides improved prediction of the timing of delivery compared to cervical length measurement alone, and this finding could have implications for patient management.

## Supplementary Information

Supplementary Information 1.

Supplementary Information 2.

Supplementary Information 3.

Supplementary Information 4.
