# Supplementary material for: The amniotic fluid proteome predicts imminent preterm delivery in asymptomatic women with a short cervix
Source: Sci Rep. 2022 Jul 11;12:11781. doi: 10.1038/s41598-022-15392-3 (PMC9276779; doi:10.1038/s41598-022-15392-3)
Supplement: Supplementary file 1 — Supplementary Information 1. [file 41598_2022_15392_MOESM1_ESM.docx]

**Supplementary Table and Figure Legends**

Supplementary Table S1. Results of biological process enrichment analysis for the association between amniotic fluid proteins and preterm birth within two weeks of amniocentesis.

Supplementary Figure S1. Distribution of gestational age at amniocentesis and delivery in the study population.

Supplementary Figure S2. ROC curves for predicting imminent delivery: a comparison between cervical length alone and models that combine cervical length with IL-6 quantified by Enzyme-Linked Immunosorbent Assay (ELISA) vs. the multiplex (aptamer-based) platform. AUC: area under the ROC curve and 95% confidence interval. CL: cervical length.
