# Supplementary figures and images for: The amniotic fluid proteome predicts imminent preterm delivery in asymptomatic women with a short cervix
Source: Sci Rep. 2022 Jul 11;12:11781. doi: 10.1038/s41598-022-15392-3 (PMC9276779; doi:10.1038/s41598-022-15392-3)

Patients

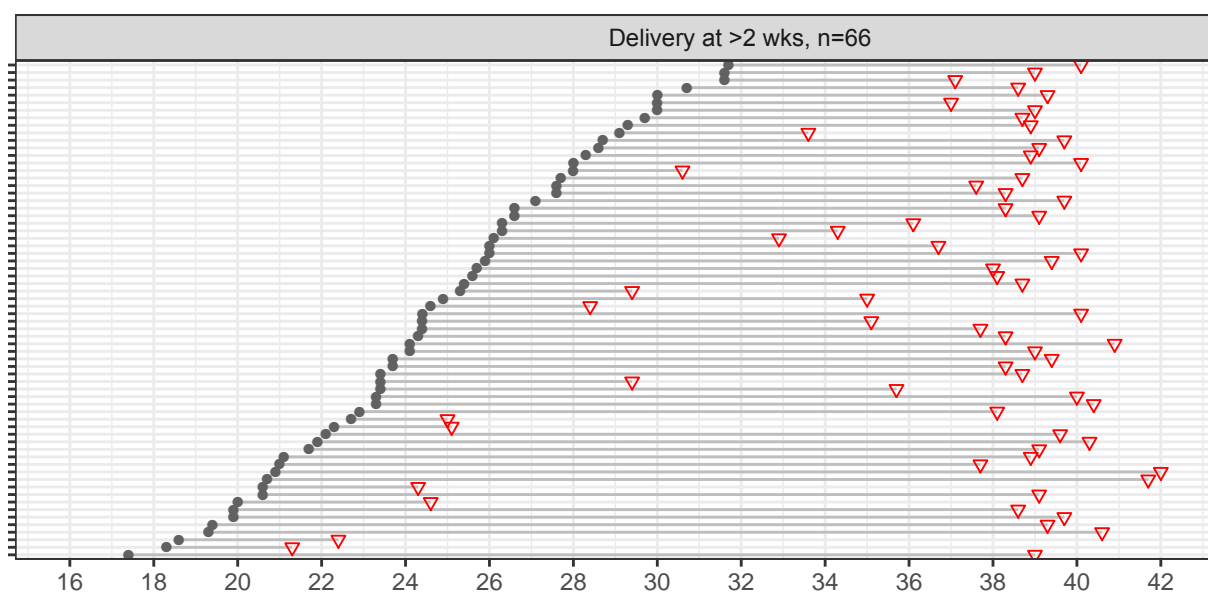

Patients

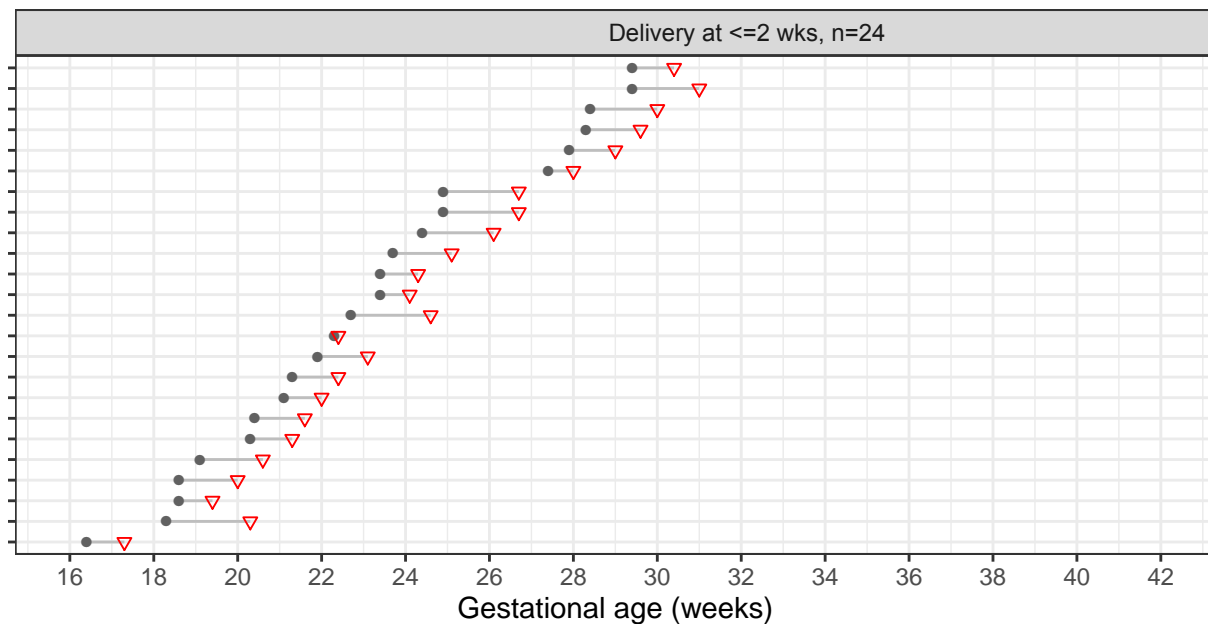

20432

● Gestational age at sample ▼ Gestational age at delivery

Supplement: Supplementary file 2 — Supplementary Information 2. [file 41598_2022_15392_MOESM2_ESM.pdf]

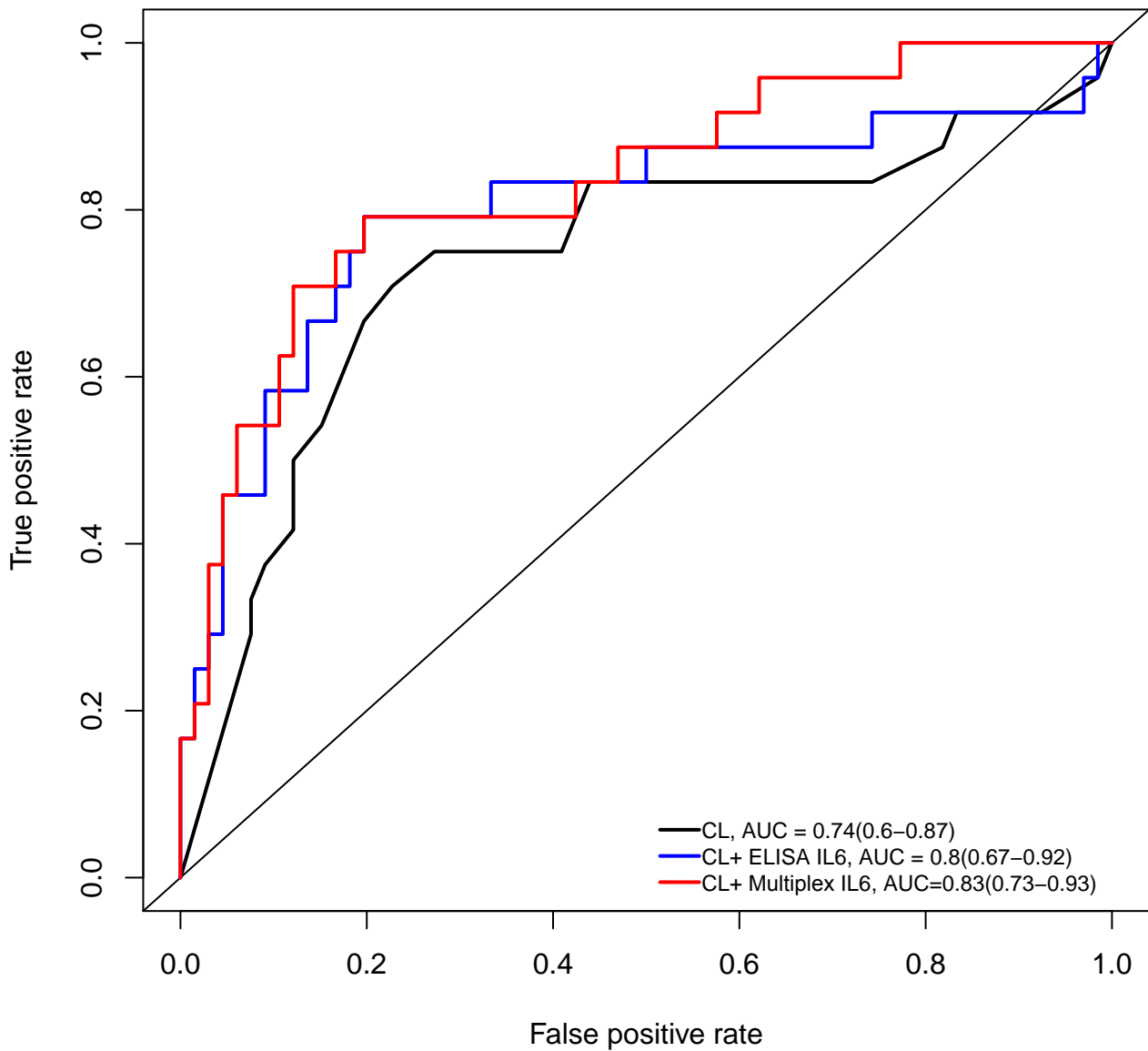

Supplement: Supplementary file 3 — Supplementary Information 3. [file 41598_2022_15392_MOESM3_ESM.pdf]
